# High-Frequency 64-Element Ring-Annular Array Transducer: Development and Preclinical Validation for Intravascular Ultrasound Imaging

**DOI:** 10.3390/bios15030169

**Published:** 2025-03-05

**Authors:** Xi Liu, Yuanlong Li, Haiguo Qin, Chang Peng

**Affiliations:** School of Biomedical Engineering, ShanghaiTech University, Shanghai 201210, China

**Keywords:** intravascular ultrasound, ring-annular array, ultrasound imaging, ultrasound transducer

## Abstract

Intravascular ultrasound (IVUS) imaging has become an essential method for diagnosing coronary artery disease. However, traditional mechanically rotational IVUS catheters encounter issues such as mechanical wear and imaging distortions in curved vessels. The ring-annular IVUS array has gained attention because it offers superior imaging performance without the need for mechanical rotational parts, thereby avoiding rotational imaging distortion. An optimized mechanical micromachining process employing precision dicing technology is proposed in this study, with the objective of achieving higher operating frequencies and minimized outer diameters for a 64-element ring-annular array. This method broadens the range of fabrication options and improves the imaging sensitivity of ring-annular IVUS arrays, as well as eliminating imaging distortion in rotational IVUS catheters, particularly in curved vessels. The probe has a 7.5 Fr (2.5 mm) outer diameter, with key fabrication steps including precision dicing, flexible circuit integration, and Parylene C encapsulation. The ring-annular array has a center frequency of 21.51 MHz with 67.87% bandwidth, with a 56 µm axial resolution and a 276 µm lateral resolution. The imaging performance is further validated by in vitro phantom imaging and ex vivo imaging.

## 1. Introduction

Intravascular ultrasound (IVUS) is an important medical imaging technology that provides high-resolution cross-sectional images of blood vessels; it is primarily used for the real-time evaluation of vessel narrowing [1]. It is widely applied in the assessment of coronary artery atherosclerosis, as guidance for stent implantation, and in the precise measurement of intravascular lesions, playing a crucial role in the diagnosis and treatment of coronary artery diseases [2,3]. By offering detailed images of the vessel wall and lumen, IVUS helps clinicians to develop more accurate treatment plans, thereby improving the success rate of interventional procedures [4].

IVUS catheters are mainly divided into two types based on their imaging method: the mechanical rotation-type and the array-type [5]. Traditional mechanical rotational IVUS catheters achieve imaging through the rotation of a single transducer [6,7]. Although they offer high resolution, they suffer from mechanical wear and image distortion, especially when imaging in curved vascular regions, where distortion is more pronounced [8,9,10]. In recent years, ring-annular IVUS catheters have gained market attention due to their high frame rates, lack of mechanical moving parts, and distortion-free imaging performance in curved vascular regions [11].

The core significance of the ring-annular IVUS catheter lies in its multi-element design, which allows for rapid imaging through the electronic switching of the elements, avoiding the issues caused by mechanical rotation [12]. The 20 MHz 64-element ring-array design strikes a good balance between resolution and penetration depth, making it suitable for the high-definition imaging of vessels such as coronary arteries. While powder/solution composite piezoelectric materials exhibit lower sensitivity than sheet materials [13], their compatibility with the precision dicing process, a key step in mechanical micromachining, offers distinct advantages. Specifically, precision dicing enables the testing of structures with a high depth-width ratios at low costs and with high efficiency [14,15]. Moreover, a key advantage of traditional cutting processes is flexibility in fabrication, which can accommodate various transducer designs with different frequencies and sizes. Recent studies have made considerable advancements in the fabrication techniques used for ultrasonic array catheters, contributing to their enhanced performance and broader clinical applications. For instance, a backing-layer-shared miniature dual-frequency ultrasound probe has been proposed, balancing imaging depth and resolution. The advantages of this catheter were demonstrated in both in vitro and ex vivo validations [16]. Additionally, a dual-mode miniature ultrasound probe for combined IVUS Doppler flow and imaging studies has been introduced, demonstrating the expanded potential of the multimodal integration of IVUS and Doppler technologies in clinical applications [17]. Ma et al. [18] proposed three prototypes of dual-frequency IVUS probes with center frequencies of 35/90 MHz, 35/120 MHz, and 35/150 MHz. Junsu Lee et al. [19] developed a dual-frequency IVUS transducer consisting of three elements arranged side by side in the horizontal (i.e., elevation) direction.

Significant advancements have also been made in micromachining techniques for endoscopic linear and ring arrays. Light et al. [20] developed two new manufacturing methods, utilizing Tyco Electronics’ braided wiring technology and custom long flexible circuits, successfully constructing arrays with 55 and 70 transducer elements and achieving the high-quality three-dimensional imaging of intravascular structures. Bezanson et al. [21] developed a miniaturized, 40-MHz, 64-element phased-array transducer (2.5 mm × 3.1 mm) using a 0.68Pb(Mg_1/3_Nb_2/3_)O_3_-0.32PbTiO_3_ (PMN-32%PT) single-crystal wafer. By wire bonding the array elements to a polyimide flexible circuit board, they achieved high-resolution imaging suitable for endoscopic applications, which was validated via ex vivo tissue imaging. Jiabing Lv et al. [22] developed a one-dimensional phased array operating at 26.2 MHz with a pitch of 32.5 µm, successfully integrating both the acoustic probe and interventional device within a single catheter and maintaining an outside diameter of 3 mm. Claire Thring et al. [23] efficiently fabricated a 32-element cylindrical array at 20 MHz with a pitch of 260 µm using a photolithography fabrication process. Furthermore, Dan et al. [24] developed a 64-element ring-shaped ultrasound transducer with a central frequency of 6.91 MHz using a PMN-PT single crystal/epoxy 1–3 composite. This method cuts without cutting through the backing layer and then wraps the array around the metal tube. Each element is connected individually via a coaxial cable, with the transducer featuring an outer diameter of 10 mm and an inner diameter of 6 mm. Zhang et al. [25] designed and implemented a 1.5D ultrasound ring array. Compared to a 1D array, a 1.5D array offers the advantage of enabling dynamic elevational focusing; a 1.5D array can also achieve dynamic elevation focusing with fewer array elements than a 2D array. Using this method, the transducer stack was cut and injected with epoxy, and then the array was connected with FPC. This ring array consisted of 84 × 5 elements with a central frequency of 6.47 MHz and an outer diameter of approximately 10 mm. The existing ultrasonic annular imaging arrays manufactured via a mechanical micromachining process have a diameter too large to be used in the IVUS imaging field.

This study aims to further reduce the size and increase the operating frequency of the ring-annular IVUS catheter, introducing an improved mechanical micromachining technique for the intravascular imaging catheter. Increasing the working frequency of the IVUS catheter can improve the axial resolution of the imaging, improve the image quality, and allow more information about the vascular anatomy image to be obtained. Reducing the catheter size can increase the speed at which the IVUS catheter passes through the curved blood vessel. In this study, A workflow is developed for manufacturing a high-frequency IVUS array using mechanical micromachining processes based on precision dicing technology to achieve a reliable and cost-effective solution. The objective is to meet the micro-size requirements of the IVUS catheter and achieve the requirements of high imaging resolution and high frame rate of the micro-IVUS catheter in curved vascular imaging. A 64-element ring-annular array ultrasound imaging catheter, operating at a central frequency of 20 MHz, has been designed and fabricated. The catheter is designed with a diameter of 7.5 Fr (2.5 mm) and has been tested for high consistency in impedance, echo sensitivity, and echo bandwidth. In vitro phantom and ex vivo porcine coronary artery imaging tests were also conducted to validate its imaging capabilities, further confirming the feasibility of the mechanical micromachining process designed for manufacturing the 64-element ring-annular IVUS array catheter.

## 2. Materials and Methods

### 2.1. Transducer Design

**Figure 1:**
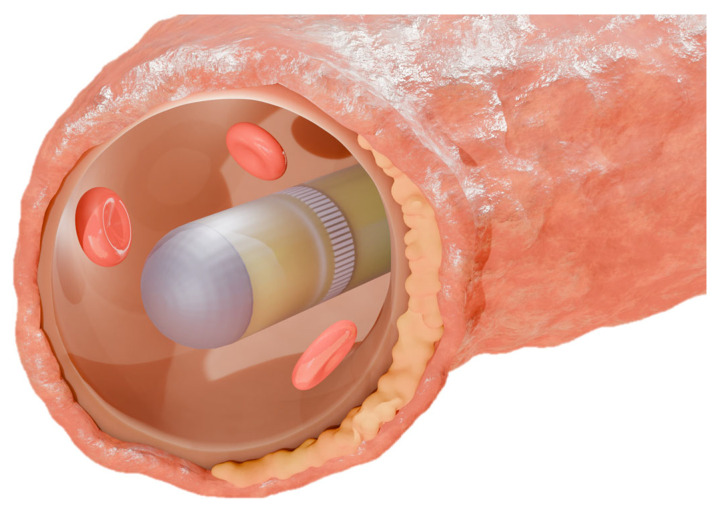
Schematic diagram of the proposed 64-element ring-annular array.

The design of the proposed IVUS transducer is guided by two main constraints: (1) the outer diameter should be minimized, as clinically used catheters typically have a maximum rigid outer diameter of 4 mm, with the final design in this study expected to stay below this size [26]. (2) The imaging penetration depth is expected to exceed 5 mm to ensure sufficient visualization [19]. As previously described, the appearance of the 64-element ring-annular IVUS array is illustrated in Figure 1.

Piezoelectric materials such as PMN-PT or PMN-PIN-PT, known for their high echo sensitivity, were not selected due to their fragility, which limits their commercial viability. Instead, PZT-5H ceramic (3203 HD, CTS, Albuquerque, NM, USA) was selected due to its superior stability, mature manufacturing process, and relatively low cost [27]. To further enhance the imaging quality, a two-layer matching layer design was adopted. To achieve optimal acoustic impedance matching, the acoustic impedance of the two layers should satisfy Equations (1) and (2) [28]. The acoustic impedance of PZT-5H is 35.3 MRayl, while the human tissue impedance is approximately 1.5 MRayl. Thus, the calculated acoustic impedances for the two matching layers are 9.12 MRayl and 2.35 MRayl. A mixture of Ag/Epoxy (Ag: Epoxy = 2.4:1, 6.3 MRayl) and Parylene C were selected as the first and second matching layers, respectively. Parylene C is a strong, resistant polymer with low acoustic impedance (2.59 MRayl). The backing layer material requires a significant acoustic attenuation (36.6 dB/cm/MHz) and a sufficient impedance mismatch with the piezoelectric material to direct more ultrasonic energy forward and absorb the rearward ultrasound, thereby achieving optimal imaging quality. Therefore, E-Solder 3022 (Von Roll USA Inc., Schenectady, NY, USA) was chosen for the backing layer.

(1) $$Z_{m1}={\left(Z_{p}^{4}\times Z_{1}^{3}\right)}^{1/7}$$

(2) $$Z_{m2}={\left(Z_{p}^{1}\times Z_{1}^{6}\right)}^{1/7}$$

The design materials and thicknesses of each transducer layer result from the Krimholtz–Leedom–Matthaei (KLM) model, and the key parameters of the materials selected for each layer are listed in Table 1. The grinding error of ±1 µm should be considered in the design, and the transducer manufactured within the target thickness and error range can achieve high performance.

**Figure 2:**
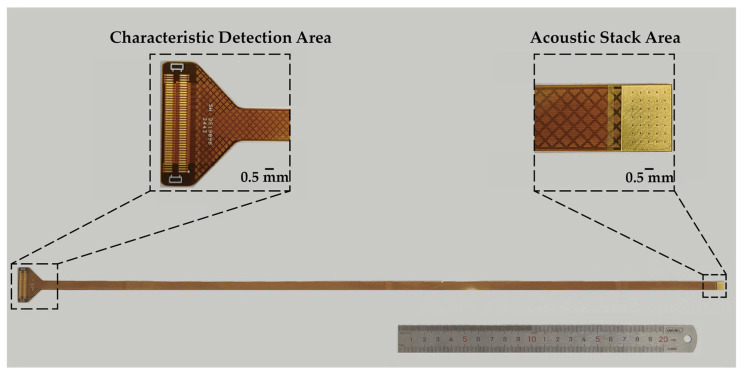
Custom-designed FPC board of the 64-element ring-annular array transducer.

Figure 2 shows the flexible printed circuit (FPC) used for assembling the acoustic stack. In the acoustic stack, the array pitch is 100 µm, the width is 50 µm, and a total of 64 array elements are arranged horizontally. The characteristic detection area is designed to weld the board-to-board interface to ensure the stable connection of the multi-array leads during switching. In this work, the final outer diameter of the outside housing is about 7.5 Fr (2.5 mm).

### 2.2. Transducer Fabrication

#### 2.2.1. Transducer Stack Fabrication

First, a Cr/Au (500/1000 Å) electrode was deposited on one surface of the PZT-5H via magnetron sputtering to form the electrode surface. E-Solder 3022 (Von Roll USA Inc., Schenectady, NY, USA) was then coated onto the electrode surface as the acoustic backing layer. Precision grinding reduced the piezoelectric layer and backing layer to thicknesses of 114 µm and 150 µm, respectively. A Cr/Au (500/1000 Å) electrode was then deposited on the other surface of the PZT-5H. The acoustic matching layer, comprising Epo-Tek 301 epoxy (Epo-Tek 301, Epoxy Technologies, Billerica, MA, USA) blended with 2–3 µm silver particles at a 1:2.4 mass ratio, was coated onto the metal electrode surface. Post-curing mechanical polishing achieved a uniform 20 µm matching layer thickness. The piezoelectric layer and the matching layer of the high-frequency transducer are thin, so the grinding step in the piezoelectric layer and the matching layer requires the use of high-precision (±1 µm) grinding equipment to ensure the accuracy of the thickness. Finally, the sample was diced into strips with an 0.4 mm × 8 mm transducer using a wafer dicing saw (DAD323, DISCO, Tokyo, Japan).

#### 2.2.2. Ring-Annular IVUS Array Assembly Fabrication

**Figure 3:**
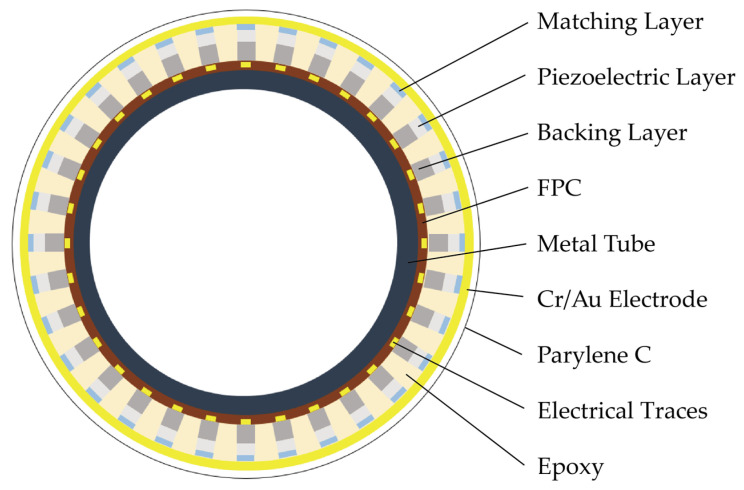
Schematic cross-section of the 64-element ring-annular array.

**Figure 4:**
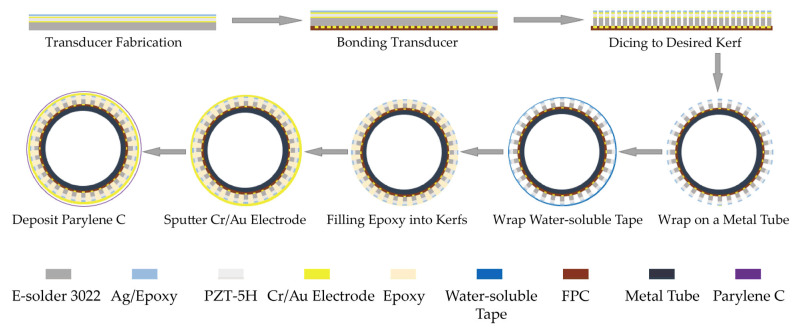
The integration workflow of the 64-element ring-annular array.

Figure 3 presents a cross-sectional view of the 64-element ring-annular array. The fabrication of the array utilized mechanical micromachining techniques. A strip with the dimensions 0.4 mm × 8 mm × 0.284 mm was initially prepared and then employed to create the ring-annular array. The fabrication steps are outlined in Figure 4. Initially, an E-Solder 3022 (Von Roll USA Inc., Schenectady, NY, USA) was used to secure the strip-shaped transducer elements onto the corresponding pads of the customized FPC (thickness ~100 µm), ensuring electrical connectivity to the backing layer. A 25 µm thick dicing saw was subsequently used to separate the electrical connections between the transducer elements. It is essential to carefully control the blade height during the dicing to ensure proper alignment with the surface of the FPC, guaranteeing the correct separation of the array elements. After dicing, the FPC was wrapped around a metal tube with a 1.8 mm outer diameter. Epo-Tek 301 was applied to the tube’s surface, and the FPC was tightly conformed around the tube using a clamp. The FPC was wrapped around the metal tube before Epo-Tek 301 was injected to achieve a smaller outer diameter of the tube. The assembly was then placed in an oven at 45 °C for 6 h to cure. Non-conductive Epo-Tek 301 was injected into the kerfs to provide insulation between the top and bottom electrodes. To protect the transducer’s front surface, water-soluble tape was applied during this process. Finally, the probe was sputtered to ground the matching layer surface, and the complete device was encapsulated using a Parylene C vacuum deposition system.

### 2.3. Characterization of the 64-Element Ring-Annular Array

After the 64-element ring-annular array was fabricated, its impedance spectrum was tested using an impedance analyzer (E4990A, Keysight Technologies, Santa Rosa, CA, USA) to verify the consistency of its impedance performance. The electromechanical coupling coefficient ($k_{t}$) was obtained using the following equation [29]:

(3) $$k_{t}=\sqrt{\;\frac{\pi }{2}\frac{f_{r}}{f_{a}}\tan[\frac{\pi }{2}\left(\frac{f_{a}-f_{r}}{f_{a}}\right)]}$$

where $f_{r}$ represents the resonance frequency and $f_{a}$ represents the antiresonance frequency.

A pulse–echo performance test was then performed on each element in degassed water, using a pulser/receiver (DPR 500, JSR Ultrasonics, Pittsford, NY, USA) for both excitation and reception. The initial excitation voltage applied by the device to the transducer was 155 V with a negative spike pulse of 125 ns. The echoes were visualized on an oscilloscope (DSOX3054G, Keysight Technologies, USA) to verify that the center frequency ($f_{c}$) and the −6 dB bandwidth (BW) of each element met the required specifications for imaging performance.

### 2.4. Imaging Evaluation

#### 2.4.1. Synthetic Aperture (SA) Ultrasound Imaging

The study conducted by Jung Woo Choe et al. [30] demonstrates that using flash imaging alone with an array-type IVUS catheter may not yield enough imaging information. Using the synthetic aperture ultrasound imaging method, one element emits ultrasound waves while all 64 elements of the array receive the reflected echoes. Sequential transmission from each of the 64 elements allows for the acquisition of 64 × 64 A-lines. The image for a field point $\vec{r}$ is calculated as follows [30]:

(4) $$I_{SA}\left(\vec{r}\right)=\sum_{i=1\;}^{64}\sum_{j=1}^{64}A_{i,j}(\tau_{i}\left(\vec{r}\right)+\tau_{j}\left(\vec{r}\right))$$

where $A_{i,j}(t)$ represents the A-line obtained from the *i*th transmission and the *j*th reception at time  t. $\tau_{i}\left(\vec{r}\right)$ and $\tau_{j}\left(\vec{r}\right)$ are the delays from the *i*th transmission and the *j*th reception to the field point $\vec{r}$.

#### 2.4.2. In Vitro Phantom and Ex Vivo Imaging

**Figure 5:**
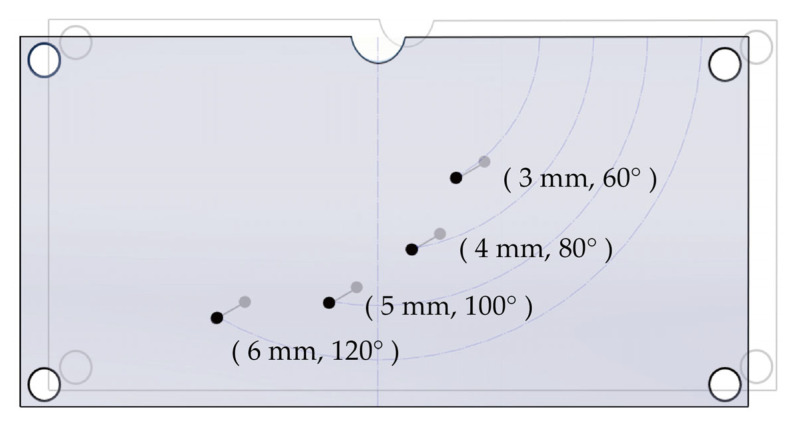
Tungsten wire imaging set-up used in the experiment (The black dots are the drilling points on the front panel).

The axial and lateral resolution tests were conducted using a tungsten wire imaging set-up with four 50 μm tungsten wires as reflectors. Holes were drilled on the acrylic phantom at positions (3 mm, 60°), (4 mm, 80°), (5 mm, 100°), and (6 mm, 120°) in the (R,θ) plane, through which the four 50 μm tungsten wires were straightened and passed. The custom acrylic phantom with tungsten wires is shown in Figure 5. The resolutions of the ring-annular array were calculated by imaging the tungsten wire imaging set-up in degassed water using the Vantage system (Verasonics Data Acquisition System, Verasonics Inc., Redmond, WA, USA). The theoretical resolution of the axial field $R_{\mathrm{axial}}$ is calculated as follows [2]:

(5) $$R_{\mathrm{axial}}=\frac{\lambda }{2\cdot BW}$$

where λ represents the wavelength of sound in the medium.

The IVUS imaging quality is represented by the signal-to-noise ratio (SNR), which is calculated as follows [2]:

(6) $$SNR=20\;\log_{10}\frac{V_{\mathrm{tissue}}}{V_{\mathrm{noise}}}$$

where $V_{\mathrm{tissue}}$ is the acoustic signal received from an echogenic region of interest, and $V_{\mathrm{noise}}$ is the signal received when no ultrasound wave is being transmitted.

**Figure 6:**
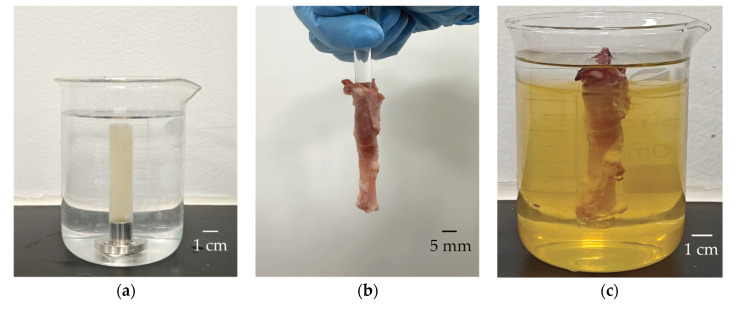
(**a**) Gelatin phantom for imaging; (**b**) porcine coronary artery extracted from a fresh pig heart; (**c**) porcine coronary artery fixed in gelatin.

The fabrication steps and proportions of the vascular phantom refer to the study conducted by Ryan et al. on the gelatin-based vascular phantom [31]. A gelatin phantom was fabricated for imaging by mixing a weight ratio of 30% gelatin (Sinopharm Chemical Reagent Co., Ltd., Shanghai, China) and a weight ratio of 2% cellulose (S3504, Sigma-Aldrich Ireland Ltd., Wicklow, Ireland) in degassed water on a heating plate with continuous stirring. The mixture was cooled to 30 °C while stirring and then poured into a custom-designed mold. After solidifying in a 4 °C refrigeration chamber for 6 h, the gelatin scattering phantom with an inner diameter of 6 mm and an outer diameter of 12 mm was removed from the mold, as shown in Figure 6a. Ex vivo porcine coronary artery imaging was also conducted in this study. As illustrated in Figure 6b, coronary arteries were extracted from a fresh porcine heart. The vessels were fixed using a weight ratio of 15% gelatin to create an ex vivo porcine coronary artery phantom for the coronary artery imaging tests.

## 3. Results

### 3.1. Manufacturing Results of the 64-Element Ring-Annular Array

**Figure 7:**
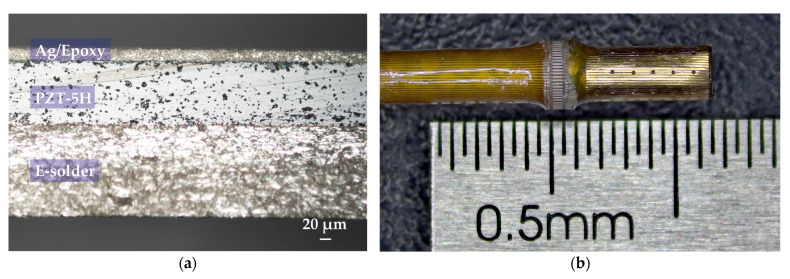
(**a**) An image of the fabricated three-layer transducer stack; (**b**) photograph of the 64-element ring-annular IVUS array before being sputter grounded.

Based on the fabrication process described in Section 2.2.1, the three-layer transducer structure is shown in Figure 7a. Subsequently, the 64-element ring-annular IVUS array, fabricated using the precision-cutting-based micromachining process designed in this study, is shown in its post-sputtered catheter form in Figure 7b.

### 3.2. Basic Performance of the 64-Element Ring-Annular Array

**Figure 8:**
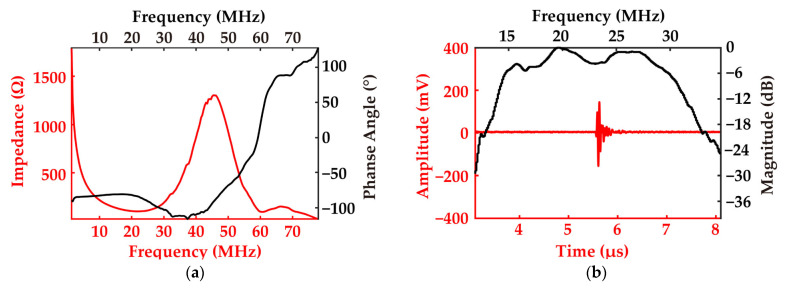
The measurements from the transducer with a 40 channel: (**a**) impedance spectrum and (**b**) pulse–echo test.

This section presents the performance test results of the 64-element ring-annular array. Figure 8a shows the impedance spectrum and Figure 8b shows the pulse–echo test of channel 40. In the pulse–echo testing, the echo originates from a metal block located 4.3 cm away from the surface of the ultrasonic transducer. The resonant frequency of the element is 21.62 MHz, and the anti-resonant frequency is 43.10 MHz. The measured pulse–echo test show a −6 dB bandwidth of 68.16% and a center frequency of 22.3 MHz.

**Figure 9:**
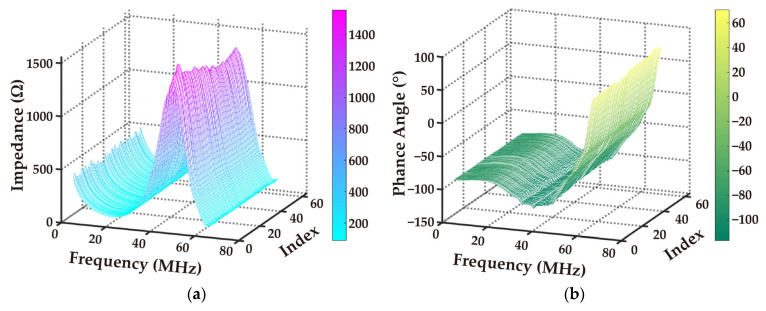
(**a**) Impedance magnitude spectrum of the 64-element array; (**b**) impedance phase spectrum of the 64-element array.

The impedance amplitude and phase spectra of the 64-element array were measured by probing two test pins connected to the impedance analyzer. These probes were switched at the pads within the characteristic detection area of the FPC. The resulting impedance spectra are shown in Figure 9.

**Figure 10:**
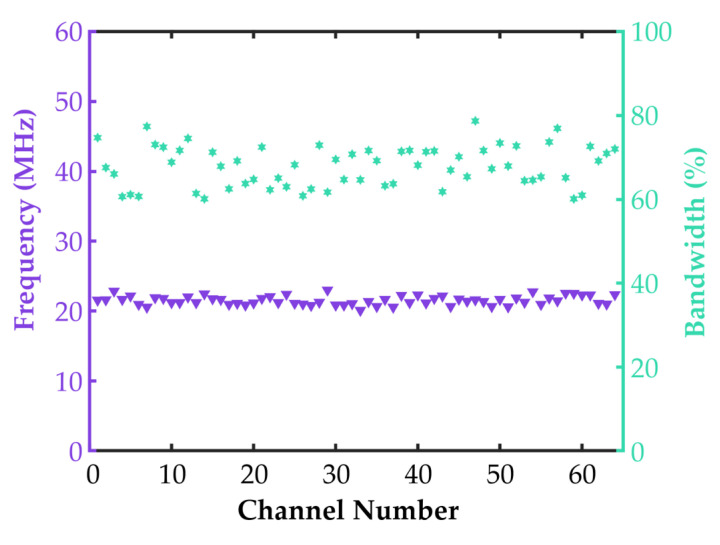
The measured center frequency and −6 dB bandwidth of the 64-element array in the pulse-echo test.

In the pulse–echo test, the center frequency and −6 dB bandwidth of the 64-element array were obtained, as shown in Figure 10. The measured center frequency and −6 dB bandwidth of the 64-element array are 21.51 ± 0.65 MHz and 67.87 ± 4.84%, respectively.

**Figure 11:**
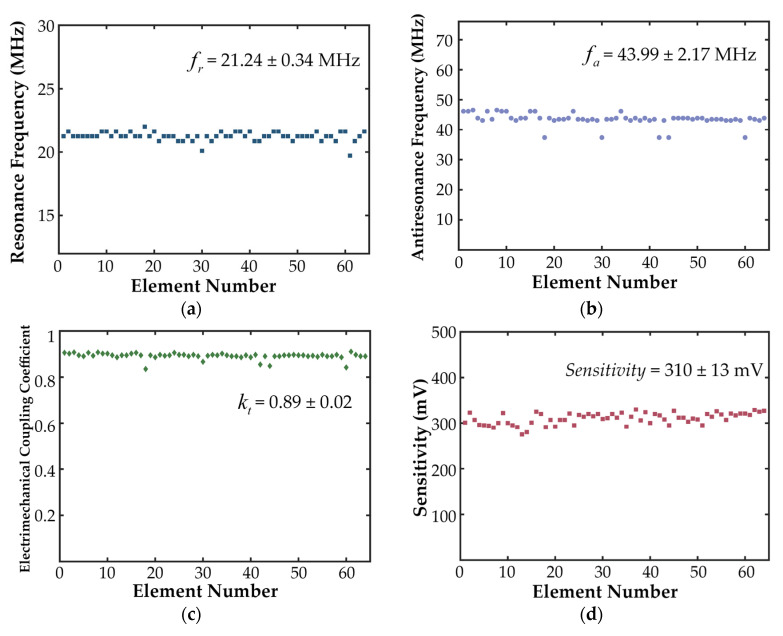
(**a**) Measured resonance frequency ($f_{r}$) of 64 elements; (**b**) measured antiresonance frequency ($f_{a}$) of 64 elements; (**c**) measured electromechanical coupling coefficient ($k_{t}$) of 64 elements; (**d**) measured peak–peak sensitivity of 64 elements.

From the impedance spectrum shown in Figure 10, the resonant frequency ($f_{r}$) and anti-resonant frequency ($f_{a}$) of the 64-element array were determined to be 21.21 ± 0.34 MHz and 43.99 ± 2.17 MHz, respectively, as illustrated in Figure 11a,b. Using Equation (3), the electromechanical coupling coefficient ($k_{t}$) was calculated to be 0.89 ± 0.02, as shown in Figure 11c. In the pulse–echo test, the measured echo sensitivity of the 64-element array was 310 ± 13 mV, as illustrated in Figure 11d.

### 3.3. In Vitro Wire Phantom Imaging

**Figure 12:**
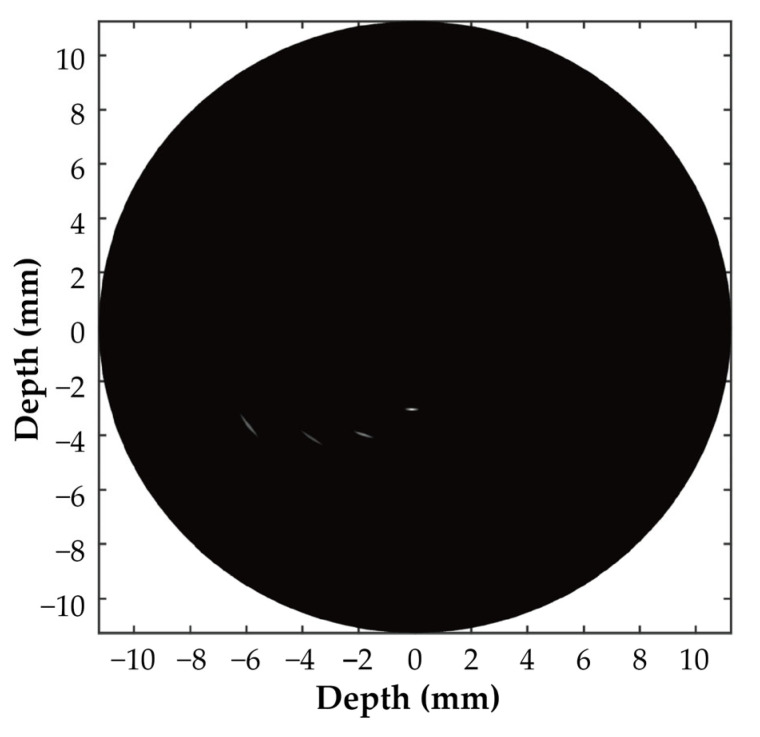
Imaging results of the tungsten wire imaging set-up.

The imaging results of the tungsten wire imaging set-up using the synthetic aperture imaging method on the Vantage data acquisition system, with a dynamic range of 40 dB, are shown in Figure 12.

**Figure 13:**
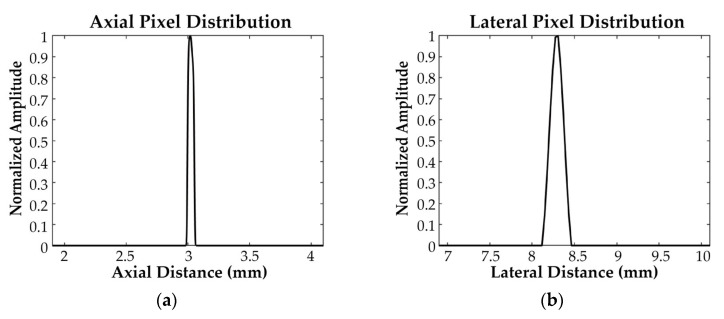
(**a**) Axial pixel distribution of the tungsten wire set-up imaging; (**b**) lateral pixel distribution of the tungsten wire set-up imaging.

An analysis of the tungsten wire set-up imaging results in Figure 11 demonstrates the axial and lateral pixel distributions of the 64-element ring-annular array, as shown in Figure 13a and Figure 13b, respectively. The measured axial and lateral resolutions of the ring-annular array were determined to be 56 μm and 276 μm, respectively.

### 3.4. In Vitro Vessel Phantom and Ex Vivo Porcine Coronary Artery Imaging

**Figure 14:**
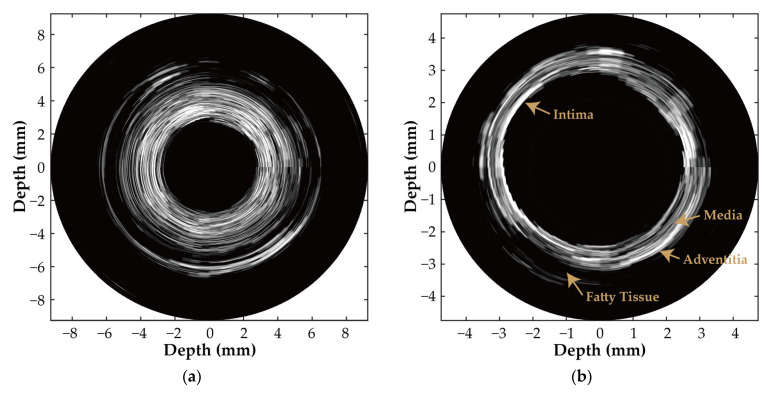
(**a**) In-vitro B-mode image acquired by the proposed 64-element ring-annular array; (**b**) ex vivo porcine coronary artery B-mode image acquired using the proposed 64-element ring-annular array.

Similarly, using the Vantage acquisition system and the synthetic aperture imaging method, the B-mode imaging results with a dynamic range of 40 dB for the vascular gelation phantom and the ex vivo porcine coronary artery phantom are shown in Figure 14a and Figure 14b, respectively. The comparison was made between the echo signal and the average signal from the non-tissue area in two different phantoms. The signal-to-noise ratio (SNR) of the gelatin vascular phantom was measured to be 28.4 dB, while the SNR of the vascular phantom was 44.1 dB. The inner diameter of the vascular phantom, which is clearly observed in Figure 14a, is 6 mm, and the outer diameter is 12 mm. In Figure 14b, the intima, media, adventitia, and fatty tissue of the vessel can be observed.

## 4. Discussion

### 4.1. Performance of the 64-Element Ring-Annular Array

The 64-element ring-annular array fabricated using the mechanical micromachining process designed in this study has a resonant frequency ($f_{r}$) of 21.21 ± 0.34 MHz, an anti-resonant frequency ($f_{a}$) of 43.99 ± 2.17 MHz, and an electromechanical coupling coefficient ($k_{t}$) of 0.89 ± 0.02. In the pulse–echo test, the echo sensitivity was measured to be 310 ± 13 mV. The 64-element ring-annular IVUS array exhibited excellent and consistent performance in terms of impedance, the electromechanical coupling coefficient, and pulse–echo sensitivity. The measured center frequency of 21.51 MHz and the −6 dB bandwidth of 67.87% indicate that the array is capable of high-resolution imaging, which is essential for IVUS applications. The axial and lateral resolutions of 56 µm and 276 µm, respectively, further confirm the array’s ability to provide detailed images of vascular structures. By calculating Equation (5), the theoretical axial resolution in degassed water (speed of sound = 1500 m/s) was determined to be 51 µm. Comparing this with the measured results, it is evident that the probe fabricated using the processing workflow designed in this study exhibits imaging performance close to the theoretical value. The in vitro phantom and ex vivo porcine coronary artery imaging results demonstrated the high imaging quality of the 64-element ring-annular array, indicating the effectiveness of the precision dicing technology employed in this study.

### 4.2. Comparison with Related Research

This study employed a mechanical micromachining process to fabricate a 64-element ring-annular IVUS array. As shown in Table 2, based on a comparison of the manufacturing results of ring-annular ultrasound arrays in related research, this study achieved both a higher operating frequency and smaller catheter dimensions. In contrast to with existing mechanical micromachining processes for manufacturing ring-annular ultrasound arrays, this study utilized FPC to replace the coaxial cables and optimized the process flow before warping the transducer stack to the metal tube, significantly enhancing circuit flexibility. These improvements enabled the catheter to accommodate 64 array elements while substantially reducing its outer diameter. During the design and fabrication of the high-frequency transducer stacks, the grinding error (±1 µm) is considered, with design parameters selected from ranges demonstrating stable high-frequency transducer stack performance within this error margin. Process adjustments were implemented during grinding and other fabrication steps to ensure stability. Ultimately, a 64-element, 20 MHz IVUS array with a 7.5 Fr (2.5 mm) diameter was successfully realized.

### 4.3. Limitations and Future Work

While the results of this study are promising, there are still some limitations that need to be addressed. The current design of the ring-annular array is limited to an outer diameter of 7.5 Fr (2.5 mm), which meets the size constraints (outer diameter < 4 mm) for the designed IVUS catheter and also achieves the smallest outer diameter for an IVUS ring catheter produced using mechanical micromachining processes. Commercially available IVUS ring catheters typically have a size of about 3 Fr. The echo sensitivity of ring catheters manufactured using the powder/solution composite piezoelectric materials in commercial IVUS catheters is lower than that attained via the mechanical micromachining process used in this study. However, the IVUS ring catheter produced in this study may not be suitable for detecting conditions in smaller blood vessels. Future work will focus on further miniaturizing the array while maintaining or improving its imaging performance. Additionally, long-term stability and durability tests will be conducted to ensure the reliability of the array in clinical settings.

## 5. Conclusions

This study further increases the frequency of the mechanical micromachining process used to produce ring-annular arrays and reduces the catheter size, resulting in the rapid fabrication of a 64-element ring-annular imaging array. Based on the proposed workflow and transducer parameters, a 64-element ring-annular ultrasound imaging catheter with an outer diameter of 7.5 Fr (2.5 mm) was successfully fabricated for IVUS applications. In transducer performance testing, the array demonstrated a resonant frequency ($f_{r}$) of 21.21 ± 0.34 MHz, an anti-resonant frequency ($f_{a}$) of 43.99 ± 2.17 MHz, and an electromechanical coupling coefficient ($k_{t}$) of 0.89 ± 0.02, with a center frequency and −6 dB bandwidth of 21.51 ± 0.65 MHz and 67.87 ± 4.84%, respectively. The imaging performance tests revealed an axial resolution of 56 µm and a lateral resolution of 276 µm in tungsten wire phantom imaging, showcasing excellent performance. Furthermore, in both the vessel phantom imaging and ex vivo vascular imaging experiments, the 64-element ring-annular array probe clearly captured the cross-sectional features of the phantoms. In conclusion, the proposed fabrication process is cost-effective and feasible, demonstrating the potential of using precision-dicing-based mechanical micromachining processes for manufacturing 64-element ring-annular IVUS arrays. This approach not only expands the fabrication methods available for IVUS arrays but also provides a low-cost and reliable technical foundation for high-quality intravascular ultrasound imaging.

## Figures and Tables

**Table 1:** Key parameters of the transducer.

Layers	Materials	Acoustic Impedance (MRayl)	Thickness (µm)
Piezoelectric	PZT-5H	35.3	114 µm
Backing	E-solder	5.92	150 µm
1st Matching	Ag/Epoxy	6.3	20 µm
2nd Matching	Parylene C	2.59	4 µm

**Table 2:** Comparison of the ring-annular array ultrasound transducers.

	Element Numbers	Frequency	Outer Diameter
Dan et al. (2011) [24]	64	6.91 MHz	10 mm
Zhang et al. (2021) [25]	64	6.47 MHz	10.4 mm
Thring et al. (2023) [23]	32	20 MHz	2.67 mm
This study	64	21.51 MHz	2.5 mm

## Data Availability

Data are contained within the article.
